# A Substrate-Dependent Bifunctional Dioxygenase from *Fraxinus chinensis* for *O*-Demethylation and C8-Hydroxylation of Coumarins

**DOI:** 10.3390/molecules31111787

**Published:** 2026-05-22

**Authors:** Xue-Ping Kong, Xue-Qing Zhong, Hong-Ling Yan, Zhuo-Zheng Xu, Jia-Xu Qin, Jing Yang, Qing-Li He, Qun-Fei Zhao

**Affiliations:** State Key Laboratory of Discovery and Utilization of Functional Components in Traditional Chinese Medicine, Innovation Research Institute of Traditional Chinese Medicine, Shanghai University of Traditional Chinese Medicine, Shanghai 201203, China; 22023638@shutcm.edu.cn (X.-P.K.); xueqingzhong@shutcm.edu.cn (X.-Q.Z.); yan_hlhl@163.com (H.-L.Y.); xuzhuozheng1997@gmail.com (Z.-Z.X.); qinjiaxu2020@163.com (J.-X.Q.); 22024727@shutcm.edu.cn (J.Y.)

**Keywords:** *Fraxinus chinensis* Roxb., 2-oxoglutarate-dependent dioxygenase, substrate-dependent bifunctionality, coumarins, *O*-demethylation, hydroxylation

## Abstract

*Fraxinus chinensis* Roxb. (Qinpi), a traditional Chinese medicinal plant, accumulates abundant coumarins that contribute to its anti-inflammatory and other bioactivities. However, the enzymatic basis for coumarin structural diversification in this species remains largely unexplored. Here, through transcriptome-wide identification of the 2-oxoglutarate-dependent dioxygenase (2OGD) family in *F. chinensis*, followed by phylogenetic analysis, heterologous expression, and in vitro enzyme assays, we identified FcDOH2, a member of the DOXC31 subfamily, which exhibits substrate-dependent bifunctionality, catalyzing the C6-*O*-demethylation of scopoletin (**3**) to esculetin (**2**) and the C8-hydroxylation of umbelliferone (**1**) to daphnetin (**6**). Using AlphaFold3-based structural modeling, molecular docking, and alanine scanning mutagenesis, we revealed that residues R155 and R221 are essential for both activities through stabilizing hydrogen bonds, whereas residue F312 acts as a functional switch, being critical for demethylation but negatively regulating hydroxylation. These findings uncover a rare bifunctional 2OGD with substrate-dependent catalytic plasticity, providing mechanistic insights into coumarin diversification in medicinal plants and a structural basis for future enzyme engineering.

## 1. Introduction

Plants produce over 200,000 structurally diverse secondary metabolites, a complexity that largely stems from the multifunctional nature of biosynthetic enzymes [1]. Fe(II)/2-oxoglutarate-dependent dioxygenases (2OGDs) are soluble non-heme iron enzymes and represent the second largest enzyme family in plants. Within their conserved 2OG-Fe(II)_Oxy domain (PF03171), they harbor a His_1_-X-Asp/Glu-Xn-His_2_ motif that chelates iron. This enables reactions such as hydroxylation and demethylation [2,3,4,5]. Notably, 2OGDs exhibit remarkable catalytic versatility, acting on diverse substrates to generate structurally complex molecules [6,7].

The amino acid sequences of plant 2OGD members are highly divergent and can be classified into three major categories: DOXA, DOXB, and DOXC [8]. The DOXC subfamily represents the largest and most functionally diverse group within the plant 2OGD family, playing a central role in the biosynthesis and modification of plant secondary metabolites, including flavonoids, coumarins, and alkaloids [1,9,10]. Phylogenetic analyses have revealed that DOXC proteins in land plants can be subdivided into multiple distinct clades with pronounced functional differentiation [3]. Among these, coumarin biosynthesis is primarily associated with DOXC30, while other clades such as DOXC31 are involved in the biosynthesis of alkaloids and other defense compounds [1].

Coumarins are a class of secondary metabolites widely distributed in plants, featuring a core benzopyrone structure. These compounds exhibit diverse biological activities and play crucial roles in plant growth, development, and stress response [11,12]. The core biosynthetic pathways of coumarins are well established, and key enzymes have been identified from various organisms [13]. Among these, 2OGDs are critical for coumarin modification. For instance, feruloyl-CoA 6′-hydroxylase (F6′H) and scopoletin 8-hydroxylase (S8H) are key enzymes in the biosynthesis of scopoletin and its derivatives [14,15]. Additionally, p-coumaroyl-CoA 2′-hydroxylase (C2′H) is involved in the biosynthesis of umbelliferone [16]. Similarly, the bifunctional 2OGD Ib2 from *Ipomoea batatas* has been shown to catalyze both the conversion of p-coumaroyl-CoA to umbelliferone and feruloyl-CoA to scopoletin [17]. Thus far, the known 2OGDs capable of modifying umbelliferone and scopoletin are limited to a few species, particularly medicinal plants rich in these coumarins.

**Figure 1:**
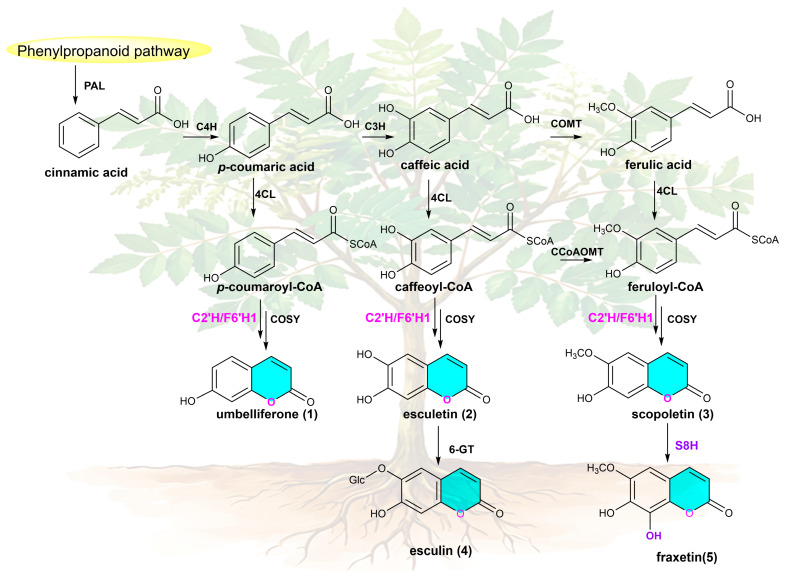
Major coumarins and their proposed biosynthetic pathway in *F. chinensis*. The structures of the major bioactive coumarins identified in *F. chinensis* are shown. The lactone ring of the coumarin scaffold is highlighted in blue. The reactions catalyzed by 2OGD enzymes involved in catalyzing different positions are distinguished in pink and purple, respectively.

*Fraxinus chinensis* Roxb. is a deciduous tree of significant medicinal importance. Its dried bark, traditionally processed into the herbal medicine known as Cortex Fraxini (Qinpi), is utilized in traditional Chinese medicine for its properties in clearing heat, drying dampness, arresting diarrhea, reducing leukorrhea, and improving vision [18,19]. Research has further demonstrated that Cortex Fraxini exhibits a broad spectrum of pharmacological activities, including anti-inflammatory [20,21], immunomodulatory [22], and anti-obesity [23,24], which are primarily attributed to its rich coumarin content, with umbelliferone (**1**), esculetin (**2**), scopoletin (**3**), esculin (**4**) and fraxetin (**5**) being the major bioactive components [15,25,26]. Recent advances in synthetic biology have enabled the heterologous production of these coumarins using engineered microbial hosts; however, the achieved titers remain low, typically in the milligram-per-liter range [13,27]. Moreover, these strategies largely depend on heterologous enzymes derived from various plant and microbial sources, and efficient biosynthetic components remain scarce. Notably, despite *F. chinensis* being a major accumulator of these coumarins, the native biosynthetic enzymes in this medicinal plant remain unexplored. Given that 2OGDs are widely involved in the structural modification and diversification of coumarins in plant secondary metabolism, this knowledge gap not only limits our understanding of coumarin diversification in *F. chinensis* but also hinders the development of more efficient biosynthetic strategies (Figure 1).

In this study, we performed transcriptome-wide identification of 2OGD genes in *F. chinensis* and functionally characterized candidate enzymes, leading to the discovery of FcDOH2, a bifunctional 2OGD with substrate-dependent demethylation and hydroxylation activities. By integrating structural modeling and site-directed mutagenesis, we explored the molecular basis for this functional duality.

## 2. Results

### 2.1. Genome-Wide Identification and Phylogenetic Analysis of 2OGDs in Fraxinus chinensis

De novo assembly of the transcriptome was performed using different tissues of *Fraxinus chinensis* Roxb., including the root, stem, leaf, and bark. In total, 135,130 transcripts and 64,839 Unigenes were generated (Tables S1 and S2). Benchmarking Universal Single-Copy Orthologs (BUSCO) assessment indicated high assembly completeness (Figure S1). To identify 2OGD family genes in *F. chinensis*, the hidden Markov model (HMM) profiles of the conserved DIOX_N (PF14226) and 2OG-FeII_Oxy (PF03171) domains were downloaded from the InterPro database. HMM searches were performed against the *F. chinensis* protein database using TBtools-II (v2.454) with a significance threshold of E-value < 1 × 10^−5^. A total of 105 candidates Fc2OGD genes were initially identified. After verifying the integrity of open reading frames (ORFs), 36 sequences that did not meet the criteria were excluded, resulting in 69 reliable Fc2OGD genes. The protein lengths of these genes ranged from 207 to 433 amino acids, with corresponding molecular weights ranging from 23.1 to 47.8 kDa (Table S3).

**Figure 2:**
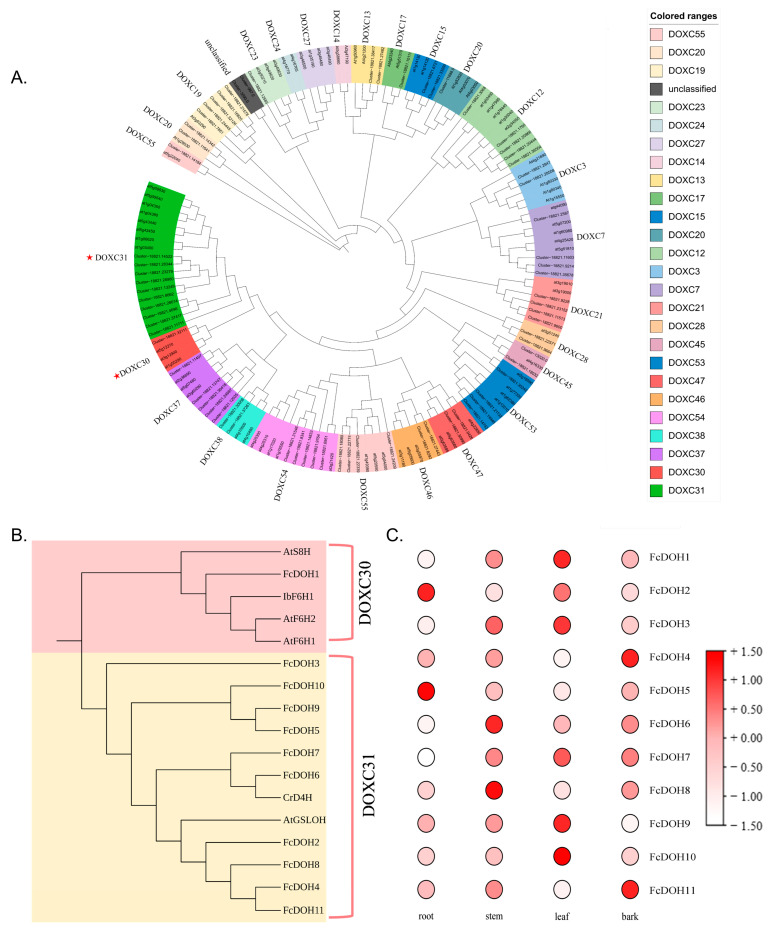
(**A**) Phylogenetic tree of Fc2OGD proteins in *F. chinensis*. The clades were functionally annotated using the 2OGDs of AtDOXC. The tree was constructed using the neighbor-joining method with 1000 bootstrap replicates. Red asterisks indicate the DOXC30 and DOXC31 clades selected for further study. (**B**) Focused phylogenetic tree of the DOXC30 and DOXC31 clades. The tree was constructed using the neighbor-joining method with 1000 bootstrap replicates, including functionally characterized enzymes from these clades together with their counterparts identified in *F. chinensis*. (**C**) FPKM-based expression profiling revealed distinct tissue-specific patterns among the Fc2OGDs.

To determine the subfamily classification of these candidate genes, phylogenetic analysis was performed using functionally characterized 2OGD proteins from *Arabidopsis thaliana* as references (Table S4). The identified Fc2OGD genes were classified into various subfamilies associated with diverse metabolic pathways, including gibberellin biosynthesis (DOXC3, DOXC7, DOXC22) and catabolism (DOXC12, DOXC13), auxin metabolism (DOXC15), glucosinolate metabolism (DOXC20, DOXC31), coumarin biosynthesis (DOXC30), salicylic acid catabolism (DOXC38), alkaloid biosynthesis and modification (DOXC31, DOXC41, DOXC52), flavonoid biosynthesis (DOXC28, DOXC47), and ethylene biosynthesis (DOXC53) [1] (Figure 2A). The remaining subfamily clades require further functional characterization. The DOXC30 clade is known to be involved in coumarin biosynthesis, whereas the DOXC31 clade is primarily associated with the biosynthesis of defense compounds such as glucosinolates and alkaloids. In F. chinensis, very few candidate genes were identified in the DOXC30 clade, while multiple candidate genes were found in its sister clade DOXC31. Given the limited representation of the DOXC30 clade and the close evolutionary relationship between the two clades, we included both subfamily clades for functional characterization. A total of 11 candidate genes belonging to these two subfamily clades were identified (Table S5). To further resolve their evolutionary relationships in *F. chinensis*, a focused phylogenetic tree was constructed using the 11 candidate Fc2OGD sequences together with functionally characterized enzymes from these two subfamily clades (Figure 2B). The results showed that ten candidate genes (FcDOH1-10) clustered with known DOXC31 subfamily enzymes (AtGSLOH [28] and CrD4H [29]), while only FcDOH11 grouped with the DOXC30 subfamily enzymes AtS8H and F6’H [14,30]. Expression analysis based on FPKM values revealed distinct tissue-specific patterns among these candidates (Figure 2C). Notably, FcDOH11 showed the highest expression in leaves, whereas FcDOH2 and FcDOH4 were predominantly expressed in roots and bark, respectively. Given that coumarins also function as defense metabolites and the two subfamily clades are evolutionarily closely related, combined with their expression profiles, both subfamily clades were selected as candidates for functional characterization.

### 2.2. Sequence Analysis of Candidate FcDOH Proteins

To assess whether the 11 candidate FcDOH proteins possess the structural features required for 2OGD catalysis, multiple sequence alignment was performed using functionally characterized DOXC30 and DOXC31 enzymes as references (Table S5). The alignment revealed that FcDOH1–FcDOH9 contain the conserved His1-X-D/E-Xn-His2 iron-binding motif, the DSBH (double-stranded β-helix) domain, and the highly conserved R-X-S motif responsible for 2-oxoglutarate (2-OG) binding [31], which are essential for catalytic activity in the 2OGD family (Figure S2). In contrast, FcDOH10 and FcDOH11 lacked both the His_1_-X-D/E-Xn-His_2_ iron-binding motif and the R-X-S 2-OG-binding motif. Based on these observations, FcDOH10 and FcDOH11 were excluded from further analysis. The remaining nine candidates (FcDOH1–FcDOH9) all possessed the complete set of conserved motifs required for 2OGD catalytic activity.

### 2.3. Gene Cloning and Heterologous Expression of FcDOH Candidates

The open reading frames (ORFs) of the nine candidate genes (FcDOH1–FcDOH9) were amplified by PCR using the primers listed in Table S7. Among them, FcDOH8 and FcDOH9 failed to yield PCR products and were therefore excluded from further analysis. The remaining seven candidates (FcDOH1–FcDOH7) were successfully amplified and cloned into the pET-28a(+) vector for heterologous expression.

The recombinant constructs were transformed into *Escherichia coli* BL21(DE3) competent cells (Table S8). Protein expression was induced with 0.5 mM IPTG at 20 °C for 20 h, and the recombinant proteins were purified by Ni-NTA affinity chromatography. SDS-PAGE analysis revealed that five of the seven recombinant proteins (FcDOH2, FcDOH3, FcDOH4, FcDOH5, and FcDOH7) were successfully expressed as soluble His-tagged proteins, with molecular weights consistent with the predicted values (42.0–43.1 kDa). In contrast, FcDOH1 and FcDOH6 were expressed predominantly as insoluble proteins and were not used for further functional studies (Figure S3). The five soluble proteins were subsequently subjected to in vitro activity assays.

### 2.4. Functional Characterization of FcDOH Proteins

**Figure 3:**
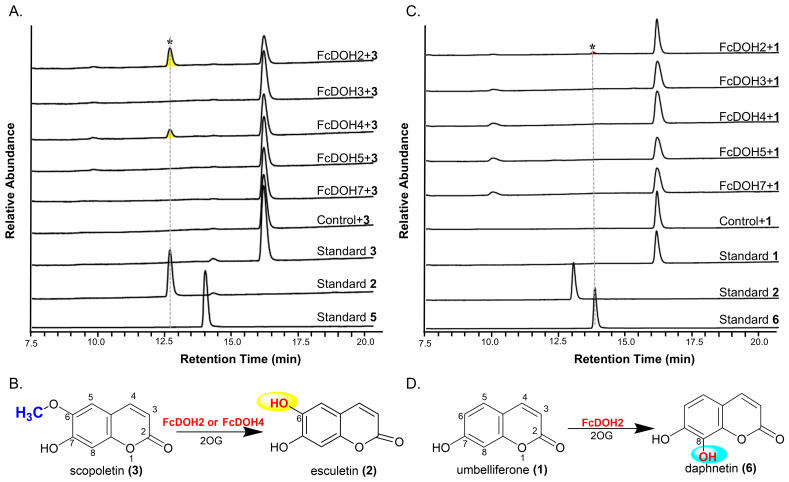
Functional characterization of candidate FcDOH enzymes. (**A**) LC-MS analysis of **3** conversion. A new product yellow peak (*) was detected in reactions with FcDOH2 and FcDOH4, identified as **2**. (**B**) Schematic representation of the C6-O-demethylation of **3** to **2** catalyzed by FcDOH2 and FcDOH4. (**C**) LC-MS analysis of **1** conversion. A new product red peak (*) was detected in the reaction with FcDOH2, identified as **6**. (**D**) Schematic representation of the C8-hydroxylation of **1** to **6** catalyzed by FcDOH2.

To evaluate the catalytic activities of the five soluble FcDOH proteins (FcDOH2, FcDOH3, FcDOH4, FcDOH5, and FcDOH7), in vitro enzyme assays were performed. Phylogenetic analysis revealed that these proteins belong to the DOXC31 clade, the sister group of the coumarin-associated DOXC30 clade (Figure 2B). Since the DOXC30 clade contains the well-characterized hydroxylase AtS8H, which catalyzes the C8 hydroxylation of scopoletin **3** to produce fraxetin **5**, we hypothesized that the DOXC31 proteins might possess similar hydroxylation activity. To test this hypothesis, scopoletin **3** was first used as a substrate. LC-MS analysis revealed that FcDOH2 and FcDOH4 converted **3** to a new product (Figure 3A). Unexpectedly, the product exhibited a [M−H]^−^ ion at *m*/*z* 177, which is 14 Da less than that of **3** (*m*/*z* 191), instead of the 16 Da increase expected from hydroxylation, indicating a demethylation reaction. Comparison with an authentic standard confirmed the product as esculetin **2**, demonstrating that FcDOH2 and FcDOH4 catalyze the C6-O-demethylation of **3** to **2,** rather than the expected hydroxylation of **3** to **5** (Figure 3B).

To further explore whether these enzymes possess hydroxylation activity, a broader range of substrates was screened (Table S9). Among the tested substrates, only umbelliferone **1** was converted by FcDOH2 to a new product (Figure 3C). The product showed a [M−H]^−^ ion at *m*/*z* 177, which is 16 Da higher than **3** (*m*/*z* 161), suggesting a hydroxylation reaction. To determine the hydroxylation position, the product was compared with two possible standards: **2** (C6-hydroxylated) and daphnetin (**6**) (C8-hydroxylated) (Figure 3D). The product co-eluted with **6** but not with **2**, indicating that FcDOH2 catalyzes the C8 hydroxylation of **1** to **6**, although the activity was weak.

These results demonstrate that FcDOH2 exhibits substrate-dependent bifunctionality, catalyzing the C6-O-demethylation of **3** to **2** and the C8 hydroxylation of **1** to **6**—two chemically distinct reactions.

### 2.5. Structural Insights into the Bifunctional Catalytic Activity of FcDOH2

To elucidate the structural basis for the bifunctional catalytic activity of FcDOH2, the three-dimensional structure was predicted using AlphaFold3. The predicted model showed high overall confidence, with an ipTM of 0.98 and a pTM of 0.89, indicating high reliability of the global fold. With the exception of some disordered sequences at the N- and C-termini, most regions of the predicted structure exhibited high accuracy, with pLDDT > 90 in the core region, indicating high confidence in individual residues. In a few regions, pLDDT exceeded 70, but the overall protein skeleton remained intact (Figure S4). FcDOH2 shows high structural similarity with DOXC family proteins that possess demethylation and hydroxylation activities, as exemplified by thebaine 6-O-demethylase (T6ODM) from *Papaver somniferum* (PDB: 5O7Y) [32] and feruloyl-CoA 6’-hydroxylase (F6’H1) from *Arabidopsis thaliana* (PDB: 4XAE) [33], whose crystal structures have been resolved, suggesting its potential bifunctional catalytic activity (Figure S5).

**Figure 4:**
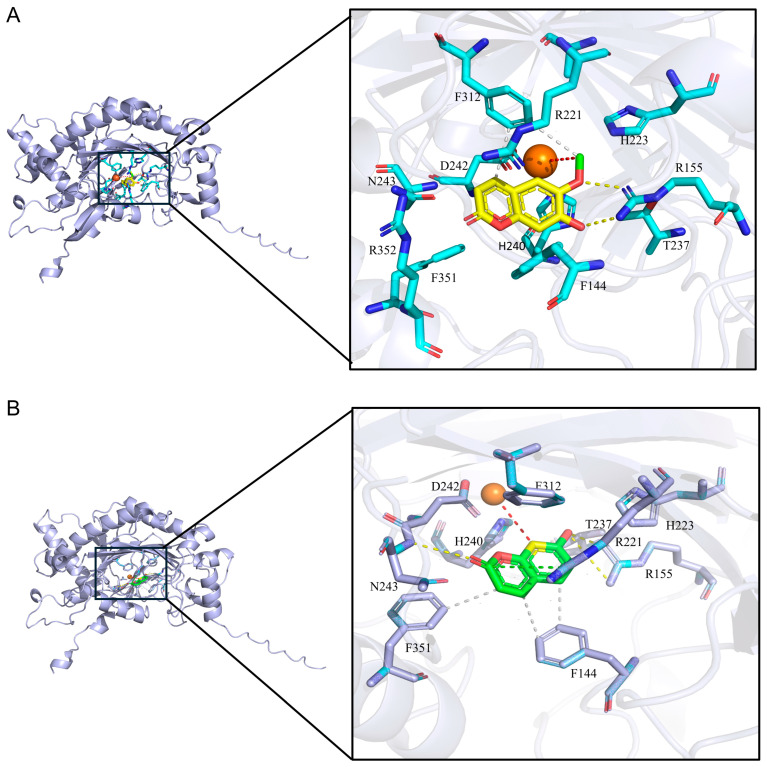
Complex models of FcDOH2 with scopoletin and umbelliferone. (**A**) Molecular docking interaction of FcDOH2 with scopoletin (yellow), with green indicating the 6-O-linked methyl group and cyan representing amino acid residues; (**B**) Molecular docking interaction of FcDOH2 with umbelliferone (green), with yellow indicating C8 and purple representing amino acid residues. Interactions are represented by dashed lines: white for hydrophobic interactions, yellow for hydrogen bonds, green for salt bridges, and red for the substrate Fe–O distance.

Molecular docking analysis revealed ten candidate residues (H240, H223, R352, F312, R155, F144, F351, D242, N243, R221, and T237) interacting with scopoletin within the active pocket of FcDOH2. R155 forms multiple hydrogen bonds with scopoletin, playing a critical role in stabilizing substrate binding. R221 also contributes to substrate stabilization via hydrogen bonds. F312 engages in hydrophobic interactions with scopoletin, providing a stable environment for the hydrophobic moiety. H240, D242, and H223 coordinate with the Fe^2+^ ion, forming the conserved H-X-D/E-Xn-H metal-binding motif characteristic of the 2OGD family. The distance between the C6 methoxy group of scopoletin and the Fe^2+^ ion falls within the favorable range for 2OGD-catalyzed reactions, indicating that scopoletin adopts a productive conformation for the demethylation reaction (Figure 4A).

For umbelliferone, ten key residues (H240, H223, F312, R155, F144, F351, D242, R221, N243 and T237) were identified, consistent with those involved in scopoletin binding, although interaction patterns differed. R155 is predicted to form hydrogen bonds with the C7 hydroxyl group of umbelliferone. Additionally, the carbonyl group of umbelliferone forms hydrogen bonds with N243, positioning the C8 atom for hydroxylation. F351 and F144 contribute hydrophobic interactions, collectively stabilizing the hydrophobic portion of the substrate. R221 forms a salt bridge with umbelliferone, further stabilizing the binding orientation. H240, D242, and H223 coordinate with the Fe^2+^ ion, constituting the conserved metal-binding motif. The distance between umbelliferone and the Fe^2+^ ion falls within the favorable range for 2OGD-catalyzed hydroxylation, indicating that umbelliferone adopts a productive conformation for the hydroxylation reaction (Figure 4B).

Based on the docking results described above, these key residues located within 4 Å of the substrates were selected as targets for alanine scanning mutagenesis to validate their functional roles in catalysis.

### 2.6. Site-Directed Mutagenesis of FcDOH2 for Functional Validation

**Figure 5:**
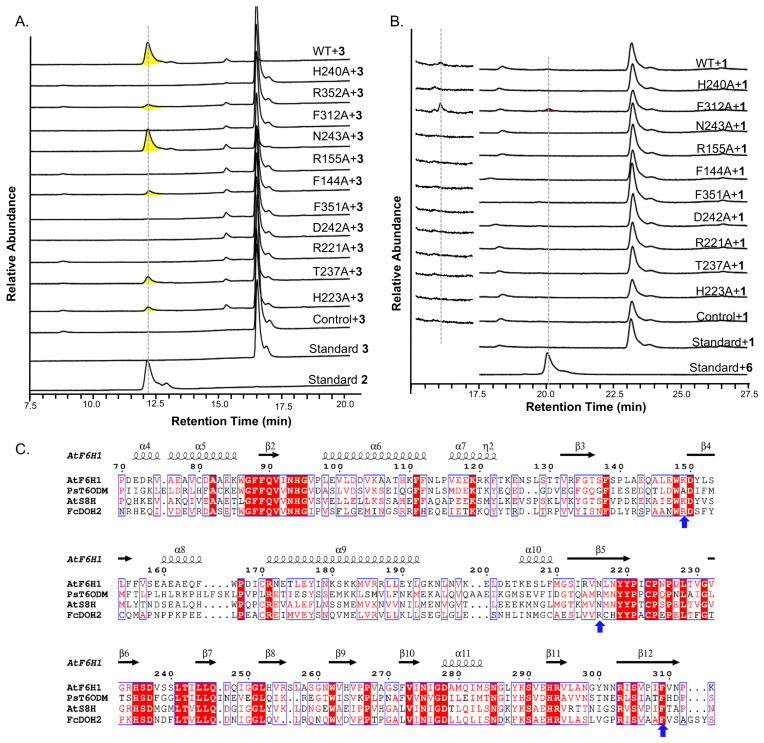
Key residues governing substrate affinity and catalysis of FcDOH2 were identified by alanine scanning mutagenesis. (**A**) UFLC analysis of alanine scanning mutants of FcDOH2 with **3**, the yellow peak indicates the product (compound **2**). (**B**) UFLC analysis of alanine scanning mutants of FcDOH2 with **1**. (**C**) Partial multiple sequence alignment of FcDOH2 with the hydroxylases AtS8H and AtF6’H1 from *Arabidopsis thaliana*, and the demethylase PsT6ODM from *Papaver somniferum*. The three key residues identified by alanine-scanning mutagenesis (R155, R221, and F312) are indicated by blue arrows.

To explore the functional roles of the residues predicted by molecular docking, alanine scanning mutagenesis was performed on FcDOH2. For scopoletin (**3**) demethylation, mutations of H240, F312, R155, F351, D242, and R221 completely abolished activity (Figure 5A). In contrast, mutations of H223, F144, R352, and T237 reduced activity, while N243A retained activity comparable to the wild-type enzyme.

For umbelliferone (**1**) hydroxylation, the wild-type enzyme showed only trace, non-quantifiable activity with product levels below the limit of quantification (<0.1%, Figure 5B). Mutations of R155 and R221 completely abolished activity, identifying them as essential for this reaction as well. Notably, the F312A mutation significantly enhanced hydroxylation activity, achieving a conversion yield of 1.79%, revealing that F312 negatively regulates this reaction. All other mutations (H223A, F144A, R352A, T237A, N243A, H240A, F351A, and D242A) resulted in no detectable activity.

To investigate the differential roles of these key residues across different enzymes, we performed a partial multiple sequence alignment of FcDOH2 with the hydroxylases AtS8H and AtF6’H1, and the demethylase PsT6ODM (Figure 5C). The alignment revealed that R155 and R221 are unique to FcDOH2 and are not conserved in AtS8H, AtF6’H1, and PsT6ODM, suggesting that these residues represent lineage-specific adaptive features that enable FcDOH2 to accommodate both scopoletin and umbelliferone as substrates. In contrast, F312 is highly conserved across all four enzymes, yet it plays a dual regulatory role in FcDOH2 that is not observed in the other three enzymes.

These findings suggest that R155 and R221 are essential for both catalytic activities, while F312 appears to play a dual role, being critical for demethylation but negatively regulating hydroxylation. These findings provide a structural basis for future enzyme engineering efforts.

## 3. Discussion

In this study, we identified FcDOH2, a DOXC31 subfamily 2OGD enzyme from *Fraxinus chinensis*, as a bifunctional catalyst with substrate-dependent activity. Unlike typical DOXC30 hydroxylases such as AtS8H and AtF6’H, which mediate single hydroxylation reactions, FcDOH2 catalyzes both the C6-O-demethylation of scopoletin and the C8 hydroxylation of umbelliferone. Although demethylation and hydroxylation are the two most prevalent functions within the 2OGD family, and multifunctional 2OGD enzymes have been widely reported, a single enzyme possessing both demethylation and hydroxylation activities has not been documented to date. This rare catalytic plasticity therefore expands our understanding of functional diversification within the 2OGD family and provides new insights into the enzymatic basis of coumarin structural diversity in medicinal plants.

Phylogenetic analysis revealed that DOXC31 and DOXC30 form a sister clade, indicating a common evolutionary origin followed by functional divergence [1,14]. The DOXC30 clade contains coumarin hydroxylases F6’H and S8H [14], while the DOXC31 clade is associated with the biosynthesis of defense compounds such as benzoxazinoids and alkaloids [1]. FcDOH2, which belongs to the DOXC31 clade, exhibits catalytic activity toward coumarin substrates, expanding the functional repertoire of this subfamily. Notably, FcDOH2 showed strong demethylation activity toward scopoletin but weak hydroxylation activity toward umbelliferone, which may reflect the functional divergence between the two clades. Furthermore, FcDOH2 did not cluster with the known demethylase PsT6ODM [10], suggesting that its demethylation activity may have evolved independently.

Protein homology modeling combined with site-directed mutagenesis is an effective approach for elucidating catalytic mechanisms in plant enzymes. To clarify the structural basis for the bifunctional activity of FcDOH2, we predicted its three-dimensional structure using AlphaFold3 and performed molecular docking. Docking results showed that R155 and R221 form hydrogen bonds with both substrates, contributing to substrate stabilization, while F312 forms hydrophobic interactions only with scopoletin, with no direct interaction with umbelliferone. Alanine scanning mutagenesis further validated the function of these residues. R155A and R221A mutations completely abolished both catalytic activities, indicating that they are critical for substrate binding (Figure 5). In contrast, the F312A mutation produced differential effects: demethylation activity toward scopoletin was lost, while hydroxylation activity toward umbelliferone was significantly enhanced. F312, an aromatic hydrophobic residue, occupies a specific position within the active pocket. For scopoletin, which carries a methoxy group at the C6 position, the hydrophobic interaction with F312 serves as a critical anchor for substrate stabilization; mutation disrupts this stabilization, leading to loss of activity. For umbelliferone, which is smaller and lacks the C6 methoxy group, the large side chain of F312 creates steric hindrance, limiting substrate access to the catalytic center; mutation relieves this spatial constraint, allowing umbelliferone to adopt a more favorable conformation for catalysis, thereby enhancing hydroxylation activity. This differential regulation by a single residue reveals the molecular basis of substrate specificity in FcDOH2 and provides a new target for enzyme engineering.

## 4. Materials and Methods 

### 4.1. Plant Materials, Chemicals and Reagents

*Fraxinus chinensis* Roxb. (Qinpi) was collected from Maguan County, Wenshan Zhuang and Miao Autonomous Prefecture, Yunnan Province, China, and identified by plant taxonomist Zhang Jun from Kunming Zhifen Biotechnology Co., Ltd. (Kunming, China). The plant was separated into roots, stems, leaves, and bark. The tissues were washed with running water, immediately frozen in liquid nitrogen, and stored at −80 °C for subsequent RNA extraction and transcriptome sequencing. Transcriptome sequencing and bioinformatic analyses were performed by Novogene Co., Ltd. (Beijing, China). Restriction enzymes NdeI and HindIII were obtained from New England Biolabs (Ipswich, MA, USA). The In-Fusion^®^ HD Cloning Kit was purchased from Baise Biotechnology (Shanghai, China). High-fidelity DNA polymerase and the HiScript^®^ II 1st Strand cDNA Synthesis Kit were acquired from Vazyme Biotech (Nanjing, China). The E.Z.N.A.^®^ Plasmid Mini Kit I and E.Z.N.A.^®^ Gel Extraction Kit were supplied by Omega Bio-tek (Norcross, GA, USA). All reference standards were sourced from Tansoole (Shanghai, China). Other reagents were purchased from Sinopharm Chemical Reagent Co., Ltd. (Shanghai, China), Sangon Biotech Co., Ltd. (Shanghai, China), Oxoid Ltd. (Basingstoke, UK), or Sigma-Aldrich (St. Louis, MO, USA).

### 4.2. Strains, Plasmids and Culture Media

The plasmids and strains used in this study are listed in Table S8. The prokaryotic expression vector pET-28a(+) (kanamycin resistance) was maintained in the laboratory. *E. coli* DH5α was used for plasmid construction and amplification, and *E. coli* BL21(DE3) was used for protein expression; both strains were purchased from Sangon Biotech Co., Ltd. (Shanghai, China). Plasmid amplification and protein expression were performed in Lysogeny Broth (LB) medium (5 g/L yeast extract, 10 g/L tryptone, 10 g/L NaCl) containing 50 μg/mL kanamycin.

### 4.3. Identification and Classification of 2OGD Family Genes in Fraxinus chinensis

Based on the transcriptome data and functional annotations of *Fraxinus chinensis* provided by Novogene Co., Ltd., genome-wide identification of 2OGD family genes was performed. To identify candidate 2OGD members, the Hidden Markov Model (HMM) profiles of the conserved DIOX_N (PF14226) and 2OG-FeII_Oxy (PF03171) domains were downloaded from the InterPro database [34]. HMM searches were conducted against the *F. chinensis* protein database using TBtools-II (v2.454) [35] with an E-value threshold of 1 × 10^−5^. Candidate genes encoding short proteins (<200 amino acids) were excluded. The remaining candidate proteins were further validated using the InterPro database. Proteins containing both the 2OG-FeII_Oxy (PF03171) and DIOX_N (PF14226) domains were considered putative Fc2OGD genes.

### 4.4. Phylogenetic Tree Construction and Sequence Analysis

To elucidate the evolutionary relationships and potential functional roles of the candidate 2OGD genes, systematic sequence analysis and phylogenetic reconstruction were performed. This analysis was based on a comprehensive sequence set, encompassing all 2OGD candidate gene sequences obtained from the transcriptome data of *Fraxinus chinensis* Roxb., along with homologous sequences derived from other plant species [10,30], including AtS8H (DOXC30), AtF6’H (DOXC30), AtGSLOH (DOXC31), and CrD4H (DOXC31). First, multiple sequence alignment was performed using ESPript 3.2 [36] to analyze conserved amino acid residues. Subsequently, a phylogenetic tree containing all 2OGD sequences was constructed using MEGA 12 [37] with the neighbor-joining method and 1000 bootstrap replicates to evaluate the phylogenetic relationships between the candidate genes and known 2OGD proteins from other species.

### 4.5. Gene Amplification and Recombinant Plasmid Construction

Based on the phylogenetic analysis described above, candidate 2OGD genes from the DOXC30 and DOXC31 clades were selected for functional characterization. Following the instructions of the HiScript^®^ II 1st Strand cDNA Synthesis Kit (Nanjing, China), reverse transcription was performed using total RNA as the template. The resulting cDNA products were stored at −20 °C for subsequent use. The open reading frames (ORFs) of the candidate genes were amplified by PCR from the corresponding cDNA templates using the primers listed in Table S7. The PCR conditions were as follows: initial denaturation at 95 °C for 3 min; 35 cycles of denaturation at 95 °C for 10 s, annealing at 58 °C for 30 s, and extension at 72 °C for 1 min, followed by a final extension at 72 °C for 5 min. The PCR products were cloned into the pET-28a(+) vector according to the instructions of the In-Fusion^®^ HD Cloning Kit. All constructs were sequenced and verified by Sangon Biotech Co., Ltd. (Shanghai, China).

### 4.6. Protein Expression and Purification

The constructed recombinant plasmids were separately transformed into *Escherichia coli* BL21(DE3) competent cells. Positive clones were cultured at 37 °C in LB medium supplemented with 50 mg/L kanamycin until the OD_600_ reached 0.6–0.8. Protein expression was then induced with 0.5 mM isopropyl β-D-1-thiogalactopyranoside (IPTG) at 20 °C for 20 h. Cells were harvested, resuspended in Tris-HCl buffer (20 mM NaH_2_PO_4_, 150 mM NaCl, 10% glycerol, pH 7.4), and lysed by high-pressure homogenization. Following centrifugation (12,000 rpm, 1 h, 4 °C) to remove debris, the supernatant was incubated with Ni-NTA Sefinose Resin (C600033, Sangon Biotech) on ice for 1 h. The mixture was loaded onto a column, and bound proteins were eluted using a stepwise gradient of imidazole (50, 100, 250, and 500 mM; 5 column volumes each). Finally, the eluted protein was desalted into storage buffer (50 mM Tris, 150 mM NaCl, 10% glycerol, pH 7.4) using a PD-10 desalting column (17085101, GE Healthcare, Chicago, IL, USA).

### 4.7. Enzyme Assays

The standard reaction mixture (100 µL) contained 50 mM Tris-HCl (pH 7.4), 0.25 mM FeSO_4_, 2 mM α-ketoglutarate, 1 mM sodium ascorbate, 400 µM substrate (scopoletin or umbelliferone), and 25 µM purified FcDOH2 protein. The reaction was incubated at 37 °C for 20 h. A negative control was prepared using heat-inactivated protein (boiled for 10 min). The reaction was terminated by adding an equal volume of methanol, followed by cooling on ice for 10 min. After centrifugation at 12,000× *g* for 15 min to remove denatured protein, the supernatant was analyzed by LC-MS. For alanine scanning mutants, the reaction products were analyzed by UFLC.

### 4.8. LC-MS and HPLC Analysis

Product analysis was performed using a Shimadzu LCMS-8040 and a Shimadzu UFLC (both from Shimadzu Corporation, Kyoto, Japan). For the Shimadzu LCMS-8040, separation was carried out on a COSMOSIL π-NAP column (4.6 I.D. × 250 mm; Nacalai Tesque, Inc., Kyoto, Japan). The mobile phase consisted of water (A) and acetonitrile (B), both containing 0.1% formic acid, with a gradient program: 0–5 min, 10% B; 5–7 min, 10–15% B; 7–10 min, 15–25% B; 10–22 min, 25–40% B; 22–27 min, 40–70% B; 27–30 min, 70–100% B; 30–32 min, 100% B; 32–37 min, 100–10% B. The flow rate was 1 mL/min at 40 °C. For the Shimadzu UFLC, separation was carried out on an Agilent Zorbax SB-C18 column (5 μm, 4.6 × 150 mm; Agilent Technologies, Santa Clara, CA, USA). The mobile phase also consisted of water (A) and methanol (B) with a gradient program: 0–5 min, 10% B; 5–10 min, 10–15% B; 10–15 min, 15–40% B; 15–22 min, 40–60% B; 22–27 min, 60–100% B; 27–30 min, 100% B; 30–32 min, 100–10% B. The flow rate was 1 mL/min at 40 °C. Authentic standards were used to confirm product identities in all analyses.

### 4.9. Molecular Docking

The three-dimensional structure of FcDOH2 was predicted using AlphaFold3 [38]. via the online server (https://www.alphafold.ebi.ac.uk, accessed on 25 December 2025). Molecular docking simulations between FcDOH2 and its substrates (scopoletin **3** and umbelliferone **1**) were performed using Schrödinger Suite. Prior to docking, the protein structure was prepared by adding hydrogen atoms and assigning partial charges. Ligand structures were downloaded from the PubChem database and energy-minimized to obtain optimal conformations. The docking grid box was centered on the predicted active site of FcDOH2, defined based on the conserved 2OG-FeII_Oxy domain and the His_1_-X-Asp/Glu-Xn-His_2_ iron-binding motif. Binding poses were ranked by predicted binding free energies, and the conformation with the lowest binding energy was selected as the optimal binding mode for each ligand. Visualization and interaction analysis were performed using PyMOL version 3.1.6.1, with analysis of hydrogen bonds and hydrophobic interactions between ligands and key residues within 4 Å of the active site.

### 4.10. Site-Directed Mutagenesis in E. coli

To elucidate the catalytic function of key amino acid residues identified by molecular docking, alanine scanning mutagenesis was performed on each predicted candidate residue of FcDOH2. The candidate residues were replaced with alanine for functional analysis. Site-directed mutagenesis was carried out using the primers listed in Table S10, with the expression plasmid containing the FcDOH2 gene as the template. PCR mixtures contained the following components: 1 µL Phanta Max Super-Fidelity DNA Polymerase, 1 µL dNTP Mix (10 mM), 25 µL 2× Phanta Max Buffer, 2 µL forward primer (10 µM), 2 µL reverse primer (10 µM), 1 µL DNA template, and double-distilled H_2_O to a final volume of 50 µL. The PCR conditions were as follows: initial denaturation at 95 °C for 3 min, 35 cycles of denaturation at 95 °C for 10 s, annealing at 58 °C for 30 s, and extension at 72 °C for 6 min 30 s, followed by a final extension at 72 °C for 5 min. All mutant genes were verified by DNA sequencing.

## 5. Conclusions

In summary, we identified FcDOH2 from *Fraxinus chinensis* as a bifunctional 2OGD enzyme that catalyzes both the demethylation of scopoletin to esculetin and the hydroxylation of umbelliferone to daphnetin in a substrate-dependent manner. To our knowledge, FcDOH2 is the first plant 2OGD experimentally shown to catalyze both O-demethylation and aromatic C-hydroxylation on coumarin substrates. Structural and mutational analyses revealed that R155 and R221 are essential for both activities, whereas F312 plays a differential regulatory role critical for demethylation while negatively modulating hydroxylation. Collectively, this study expands our understanding of functional diversification within the 2OGD family and offers a promising enzymatic tool for the heterologous production of bioactive coumarins.

## Data Availability

The gene sequence and protein sequences for *Fraxinus chinensis* are deposited in the NCBI database (https://www.ncbi.nlm.nih.gov/; BioProject ID PRJNA1446208).

## Supplementary Materials

The following supporting information can be downloaded at: https://www.mdpi.com/article/10.3390/molecules31111787/s1, Table S1: Summary of transcriptome sequencing data for F. chinensis; Table S2: Assembly statistics of F. chinensis transcriptome; Table S3: Candidate Fc2OGD genes identified by HMM search in F. chinensis; Table S4: Functionally characterized 2OGD proteins from Arabidopsis thaliana used for phylogenetic analysis; Table S5: Candidate FcDOH genes identified in F. chinensis; Table S6: Functionally characterized 2OGD enzymes used for sequence alignment; Table S7: Primers used for PCR amplification of FcDOH candidate genes; Table S8: Strains and plasmids used in this study; Table S9: Activity screening of FcDOH proteins; Table S10: Primers used for site-directed mutagenesis; Figure S1: BUSCO assessment results of the F. chinensis transcriptome assembly; Figure S2: Multiple sequence alignment of candidate FcDOH proteins; Figure S3: SDS-PAGE analysis of recombinant FcDOH proteins; Figure S4: AlphaFold3-predicted structure of FcDOH2; Figure S5: Structural similarity between FcDOH2 and DOXC family proteins with similar functions.
