# Supplementary material for: A Substrate-Dependent Bifunctional Dioxygenase from Fraxinus chinensis for O-Demethylation and C8-Hydroxylation of Coumarins
Source: Molecules. 2026 May 22;31(11):1787. doi: 10.3390/molecules31111787 (PMC13258316; doi:10.3390/molecules31111787)

Supplementary Information

A Substrate-Dependent Bifunctional Dioxygenase from *Fraxinus chinensis* for O-Demethylation and C8-Hydroxylation of Coumarins

Xue-Ping Kong [1], Xue-Qing Zhong [1], Hong-Ling Yan [1], Zhuo-Zheng Xu [1], Jia-Xu Qin [1], Jing Yang [1], Qing-Li He [1,*] and Qun-Fei Zhao [1,*]

[1] State Key Laboratory of Discovery and Utilization of Functional Components in Traditional Chinese Medicine, Innovation Research Institute of Traditional Chinese Medicine, Shanghai University of Traditional Chinese Medicine, 1200 Cailun Road, Shanghai 201203, China.

* Corresponding author's address: Shanghai University of Traditional Chinese Medicine, Shanghai, China. Tel: +86-21-51322762.

E-mail: qinglihe@shutcm.edu.cn. (Q.-L.H.); qunfeizhao@shutcm.edu.cn. (Q.-F.Z.).

Abstract

Fraxinus chinensis Roxb. (Qinpi), a traditional Chinese medicinal plant, accumulates abundant coumarins that contribute to its anti-inflammatory and other bioactivities. However, the enzymatic basis for coumarin structural diversification in this species remain largely unexplored. Here, through transcriptome-wide identification of the 2-oxoglutarate-dependent dioxygenase (2OGD) family in F. chinensis, followed by phylogenetic analysis, heterologous expression, and in vitro enzyme assays, we identified FcDOH2, a member of the DOXC31 subfamily, which exhibits substrate-dependent bifunctionality, catalyzing the C6-O-demethylation of scopoletin to esculetin and the C8-hydroxylation of umbelliferone to daphnetin. Using AlphaFold3-based structural modeling, molecular docking, and alanine scanning mutagenesis, we revealed that residues R155 and R221 are essential for both activities through stabilizing hydrogen bonds, whereas residue F312 acts as a functional switch, being critical for demethylation but negatively regulating hydroxylation. These findings uncover a rare bifunctional 2OGD with substrate-dependent catalytic plasticity, providing mechanistic insights into coumarin diversification in medicinal plants and a structural basis for future enzyme engineering.

Keywords:*Fraxinus chinensis* Roxb. (Qinpi); 2-oxoglutarate-dependent dioxygenase; substrate-dependent bifunctionality; coumarins; *O*-demethylation; hydroxylation.

Contents

1. Supplementary Table 4

Table S1. Summary of transcriptome sequencing data for *F. chinensis*........................4

Table S2. Assembly statistics of *F. chinensis* transcriptome...4

Table S3. Candidate Fc2OGD genes identified by HMM search in *F. chinensis* 4

Table S4. Functionally characterized 2OGD proteins from *Arabidopsis thaliana* used for phylogenetic analysis 7

Table S5. Candidate FcDOH genes identified in *F. chinensis* 12

Table S6. Functionally characterized 2OGD enzymes used for sequence alignment. 13

Table S7. Primers used for PCR amplification of FcDOH candidate genes 13

Table S8. Strains and plasmids used in this study 14

Table S9. Activity screening of FcDOH proteins..14

Table S10. Primers used for site-directed mutagenesis 15

2.Supplementary Figures 16

Figure S1. BUSCO assessment results of the *F. chinensis* transcriptome assembly...16

Figure S2. Multiple sequence alignment of candidate FcDOH proteins. 16

Figure S3. SDS-PAGE analysis of recombinant FcDOH proteins. 18

Figure S4. AlphaFold3-predicted structure of FcDOH2...18

Figure S5. Structural similarity between FcDOH2 and DOXC family proteins with similar functions...19

1. Supplementary Table

Table S1. Summary of transcriptome sequencing data for *F. chinensis*

sample	Raw reads	Clean reads	Clean bases (G)	Q30 (%)	GC (%)
Root	20,804,416	20,381,499	6.1	92.94	44.75
Stem	19,650,291	18,884,772	5.7	93.08	44.01
Leaf	19,592,202	19,214,477	5.8	94.63	43.51
Bark	22,042,861	21,208,837	6.4	94.51	46
Total/Average	82,089,770	79,689,585	24	93.79	44.57

Note: Raw Reads: total number of raw reads; Clean Reads: number of reads after quality filtering; Clean Bases: total bases after quality filtering; Q30: percentage of bases with Phred quality score ≥30; GC: percentage of guanine and cytosine bases.

Table S2. Assembly statistics of *F. chinensis* transcriptome

Category	Transcripts	Unigenes
Total number	135,130	64,839
Total length (bp)	171,841,411	70,662,724
Mean length (bp)	1,272	1,090
Max length (bp)	16,662	16,662
N50 (bp)	1,872	1,630
N90 (bp)	556	461
GC content (%)	44.57	44.57

Table S3. Candidate Fc2OGD genes identified by HMM search in *F. chinensis*

Gene ID	Length (aa)	Molecular Weight	E-value
Cluster-13020.0	207	23.1 kDa	5.90E-31
Cluster-18821.1760	228	25.4 kDa	8.40E-08
Cluster-18821.36504	238	26.6 kDa	1.90E-17

Cluster-18821.20444	252	28.1 kDa	2.40E-07
Cluster-18821.4696	258	29.7 kDa	1.50E-34
Cluster-18821.30058	262	29.9 kDa	4.90E-36
Cluster-18821.37261	262	29.9 kDa	1.20E-35
Cluster-18821.38417	269	30.4 kDa	1.50E-13
Cluster-18821.28980	275	30.7 kDa	2.20E-21
Cluster-18821.28344	281	31.8 kDa	1.30E-20
Cluster-18821.30242	282	31.9 kDa	4.00E-28
Cluster-18821.27443	287	32.3 kDa	1.50E-21
Cluster-18821.7851	287	32.1 kDa	3.20E-11
Cluster-18821.24445	291	32.5 kDa	1.00E-07
Cluster-18821.32126	294	33.0 kDa	4.30E-09
Cluster-18821.21578	295	32.7 kDa	2.80E-09
Cluster-18821.2847	301	33.7 kDa	2.50E-34
Cluster-18821.33550	304	34.0 kDa	1.80E-22
Cluster-18821.12585	305	33.9 kDa	1.80E-20
Cluster-18821.6721	307	33.8 kDa	7.40E-21
Cluster-18821.14743	309	35.1 kDa	2.30E-34
Cluster-18821.16820	309	34.1 kDa	1.10E-09
Cluster-11688.0	316	35.5 kDa	2.30E-21
Cluster-18821.21132	318	36.1 kDa	5.60E-34
Cluster-18821.18032	321	35.9 kDa	6.90E-31
Cluster-18821.10111	321	36.1 kDa	1.70E-25
Cluster-18821.11497	324	36.9 kDa	3.10E-24
Cluster-18821.3068	325	36.8 kDa	6.60E-25
Cluster-18821.37417	331	37.9 kDa	8.80E-28
Cluster-18821.14946	332	37.3 kDa	1.40E-34
Cluster-18821.30990	335	37.8 kDa	4.90E-32
Cluster-18821.27482	339	38.7 kDa	1.60E-32

Cluster-18821.20966	339	38.1 kDa	3.00E-27
Cluster-18821.22339	343	39.6 kDa	2.20E-30
Cluster-18821.36418	345	38.8 kDa	7.40E-28
Cluster-18821.23152	349	40.0 kDa	3.40E-34
Cluster-18821.9600	349	40.0 kDa	9.80E-34
Cluster-18821.11513	349	40.3 kDa	1.10E-32
Cluster-18821.9228	349	40.0 kDa	4.20E-32
Cluster-18821.18080	352	39.5 kDa	5.20E-29
Cluster-18821.9426	353	39.8 kDa	1.10E-30
Cluster-18821.31546	354	39.9 kDa	8.10E-35
Cluster-18821.8341	354	40.1 kDa	1.40E-33
Cluster-18821.22774	355	39.9 kDa	5.30E-30
Cluster-18821.32111	357	40.0 kDa	1.60E-32
Cluster-18821.9764	361	41.0 kDa	1.90E-29
Cluster-18821.8292	363	41.3 kDa	9.40E-36
Cluster-18821.24997	363	40.7 kDa	8.60E-32
Cluster-18821.24209	363	40.9 kDa	1.70E-26
Cluster-18821.13747	364	40.7 kDa	1.60E-28
Cluster-18821.11603	364	41.1 kDa	9.40E-37
Cluster-18821.14833	365	41.8 kDa	5.70E-34
Cluster-18821.9664	367	41.4 kDa	5.00E-30
Cluster-18821.6951	369	42.0 kDa	5.90E-32
Cluster-18821.11711	370	42.0 kDa	8.60E-30
Cluster-18821.28508	371	41.4 kDa	3.00E-31
Cluster-967.0	371	41.0 kDa	1.50E-14
Cluster-3090.0	371	41.2 kDa	1.10E-14
Cluster-18821.14522	372	41.5 kDa	4.70E-32
Cluster-18821.12035	372	41.9 kDa	3.20E-32
Cluster-18821.28014	373	42.0 kDa	5.60E-31

Cluster-18821.23279	374	42.0 kDa	1.50E-31
Cluster-18821.22571	374	41.9 kDa	1.50E-29
Cluster-18821.8662	377	42.9 kDa	7.10E-30
Cluster-18821.2587	379	43.0 kDa	1.90E-33
Cluster-18821.13249	380	43.1 kDa	3.40E-31
Cluster-18821.35678	387	43.7 kDa	1.00E-35
Cluster-18821.9214	387	43.7 kDa	1.20E-34

Table S4. Functionally characterized 2OGD proteins from *Arabidopsis thaliana* used for phylogenetic analysis

Gene ID	Functional Annotation	Functional Classification
At3g63290	2OG-Fe(II) oxygenase superfamily protein	DOXC19
At4g03050	Glucosinolate biosynthesis-related	DOXC20
At1g52820	2OG-Fe(II) oxygenase superfamily protein	DOXC20
At4g03070	Glucosinolate biosynthesis-related	DOXC20
At1g28030	2OG-Fe(II) oxygenase superfamily protein	DOXC20
At4g23340	2OG-Fe(II) oxygenase superfamily protein	DOXC17
At5g51310	2OG-Fe(II) oxygenase superfamily protein	DOXC17
At3g47190	1-aminocyclopropane-1-carboxylate oxidase (ACO)	DOXC14
At5g58660	Gibberellin 2-beta-dioxygenase (GA2ox)	DOXC14

At4g21200	2OG-Fe(II) oxygenase superfamily protein	DOXC13
At1g50960	2OG-Fe(II) oxygenase superfamily protein	DOXC13
At4g21690	2OG-Fe(II) oxygenase superfamily protein	DOXC3
At1g80330	Gibberellin 3-oxidase (GA3ox)	DOXC3
At1g15550	Gibberellin 3-oxidase (GA3ox1)	DOXC3
At1g80340	Gibberellin 3-oxidase homolog	DOXC3
At1g14120	2OG-Fe(II) oxygenase superfamily protein	DOXC15
At1g14130	2OG-Fe(II) oxygenase superfamily protein	DOXC15
Atg440900	2OG-Fe(II) oxygenase	DOXC7
At1g60980	2OG-Fe(II) oxygenase superfamily protein	DOXC7
At5g07200	2OG-Fe(II) oxygenase superfamily protein	DOXC7
At5g51810	2OG-Fe(II) oxygenase superfamily protein	DOXC7
At4g25420	Gibberellin 20-oxidase (GA20ox1)	DOXC7
At1g47990	2OG-Fe(II) oxygenase superfamily protein	DOXC12
At1g02400	2OG-Fe(II) oxygenase superfamily protein	DOXC12
At2g34555	2OG-Fe(II) oxygenase superfamily protein	DOXC12
At1g30040	Gibberellin 2-oxidase (GA2ox)	DOXC12
At1g78440	Gibberellin 2-oxidase (GA2ox)	DOXC12

At3g50210	2OG-Fe(II) oxygenase superfamily protein	DOXC23
At3g49630	2OG-Fe(II) oxygenase superfamily protein	DOXC23
At3g49620	2OG-Fe(II) oxygenase superfamily protein	DOXC23
At4g16765	2OG-Fe(II) oxygenase superfamily protein	DOXC24
At4g16770	2OG-Fe(II) oxygenase superfamily protein	DOXC24
At1g35190	2OG-Fe(II) oxygenase superfamily protein	DOXC27
At3g46490	2OG-Fe(II) oxygenase superfamily protein	DOXC27
At3g46500	2OG-Fe(II) oxygenase superfamily protein	DOXC27
At3g46480	2OG-Fe(II) oxygenase superfamily protein	DOXC27
At3g19000	2OG-Fe(II) oxygenase superfamily protein	DOXC21
At3g19010	2OG-Fe(II) oxygenase superfamily protein	DOXC21
At4g10500	Salicylic acid 5-hydroxylase (S5H)	DOXC38
At4g10490	Salicylic acid 5-hydroxylase homolog	DOXC38
At2g36690	2OG-Fe(II) oxygenase superfamily protein	DOXC37
At5g07480	2OG-Fe(II) oxygenase superfamily protein	DOXC37

At3g60290	2OG-Fe(II) oxygenase superfamily protein	DOXC37
At5g12270	2OG-Fe(II) oxygenase superfamily protein	DOXC30
At3g12900	Scopoletin 8-hydroxylase (S8H)	DOXC30
At1g55290	Feruloyl-CoA 6'-hydroxylase (F6'H)	DOXC30
At5g43440	2OG-Fe(II) oxygenase superfamily protein	DOXC31
At5g43450	2OG-Fe(II) oxygenase superfamily protein	DOXC31
At5g59530	2OG-Fe(II) oxygenase superfamily protein	DOXC31
At5g59540	2OG-Fe(II) oxygenase superfamily protein	DOXC31
At1g04350	2OG-Fe(II) oxygenase superfamily protein	DOXC31
At1g04380	2OG-Fe(II) oxygenase superfamily protein	DOXC31
At1g06620	2OG-Fe(II) oxygenase superfamily protein	DOXC31
At1g03400	2OG-Fe(II) oxygenase superfamily protein	DOXC31
At3g51240	Flavanone 3-hydroxylase (F3H)	DOXC28
At4g16330	2OG-Fe(II) oxygenase superfamily protein	DOXC45
At3g55970	2OG-Fe(II) oxygenase superfamily protein	DOXC46
At3g11180	2OG-Fe(II) oxygenase superfamily protein	DOXC46

At5g05600	2OG-Fe(II) oxygenase superfamily protein	DOXC46
At4g22880	2OG-Fe(II) oxygenase superfamily protein	DOXC47
At5g63595	2OG-Fe(II) oxygenase superfamily protein	DOXC47
At5g63580	2OG-Fe(II) oxygenase superfamily protein	DOXC47
At2g19590	1-aminocyclopropane-1-carboxylate oxidase (ACO1)	DOXC53
At1g77330	2OG-Fe(II) oxygenase superfamily protein	DOXC53
At1g12010	2OG-Fe(II) oxygenase superfamily protein	DOXC53
At1g62380	2OG-Fe(II) oxygenase superfamily protein	DOXC53
At5g54000	2OG-Fe(II) oxygenase superfamily protein	DOXC55
At1g49390	2OG-Fe(II) oxygenase superfamily protein	DOXC55
At5g20550	2OG-Fe(II) oxygenase superfamily protein	DOXC55
At5g22040	2OG-Fe(II) oxygenase superfamily protein	DOXC55
At3g21420	2OG-Fe(II) oxygenase superfamily protein	DOXC55
At1g78550	2OG-Fe(II) oxygenase superfamily protein	DOXC54

At1g17020	2OG-Fe(II) oxygenase superfamily protein	DOXC54
At4g25300	2OG-Fe(II) oxygenase superfamily protein	DOXC54
At4g25310	2OG-Fe(II) oxygenase superfamily protein	DOXC54

Table S5. Candidate FcDOH genes identified in *F. chinensis*

Transcript_ID	Name	Descirption
Cluster-18821.32111	FcDOH1	feruloyl CoA ortho-hydroxylase 1-like
Cluster-18821.23279	FcDOH2	1-aminocyclopropane-1-carboxylate oxidase-like protein
Cluster-18821.11711	FcDOH3	deacetoxyvindoline 4-hydroxylase-like
Cluster-18821.14522	FcDOH4	1-aminocyclopropane-1-carboxylate oxidase homolog
Cluster-18821.28014	FcDOH5	deacetoxyvindoline 4-hydroxylase-like
Cluster-18821.8662	FcDOH6	deacetoxyvindoline 4-hydroxylase-like
Cluster-18821.13249	FcDOH7	1-aminocyclopropane-1-carboxylate oxidase homolog 1-like isoform X1
Cluster-18821.4696	FcDOH8	1-aminocyclopropane-1-carboxylate oxidase homolog 1 isoform X1
Cluster-18821.37417	FcDOH9	1-aminocyclopropane-1-carboxylate oxidase homolog 1-like
Cluster-18821.28980	FcDOH10	1-aminocyclopropane-1-carboxylate oxidase homolog 1-like
Cluster-18821.28344	FcDOH11	1-aminocyclopropane-1-carboxylate oxidase homolog

Table S6. Functionally characterized 2OGD enzymes used for sequence alignment

Gene Name	Species	Subfamily	Function	Accession
AtS8H	Arabidopsis thaliana	DOXC30	Scopoletin 8-hydroxylase	At3g12900
AtF6'H	Arabidopsis thaliana	DOXC30	Feruloyl-CoA 6'-hydroxylase	At3g13610
AtGSLOH	Arabidopsis thaliana	DOXC31	Glucosinolate/alkaloid metabolism	At2g25450
CrD4H	Catharanthus roseus	DOXC31	Deacetoxyvindoline 4-hydroxylase	AF008597

Table S7. Primers used for PCR amplification of FcDOH candidate genes

primers	Sequences(5'-3')
FcDOH1-F	gccgcgcggcagccatATGTCAGAATCACTCACTGAAC
FcDOH1-R	gtgcggccgcaagcttTCATATTTTTGCATACTCAATTGT
FcDOH2-F	ccgcgcggcagccatATGGCGGTTCCTAACATTGAG
FcDOH2-R	gtgcggccgcaagcttTCATCCATCTACATCTGGGTTC
FcDOH3-F	gtgccgcgcggcagccatATGGTGTCTACTAGAAGTGAAAAATAT
FcDOH3-R	ctcgagtgcggccgcaagcttCTATGGAATGATTCTTTCAATCTTG
FcDOH4-F	gtgcggccgcaagcttTTAGAGCCTGAAGTCCAACAAG
FcDOH4-R	ccgcgcggcagccatATGAAATCCAGCAGTTCAACTG
FcDOH5-F	ccgcgcggcagccatATGAATATGTCGACCCTCAAAG
FcDOH5-R	ctcgagtgcggccgcaagcttCTAGACTTTTAACTTGAACTCATCAAGG
FcDOH6-F	ccgcgcggcagccatATGTTCACGAGCATTCGGAAAG
FcDOH6-R	agtgcggccgcaagcttTTAGTTCTCTGGGGTCTGCTTATGG
FcDOH7-F	gtgccgcgcggcagccatATGGTGGGCACAGACTGTGG

FcDOH7-R	ctcgagtgcggccgcaagcttTCACTCATTGGCACTTGCATTC
FcDOH8-F	ccgcgcggcagccatATGCTTGATGGAATAAGAAGGTTTC
FcDOH8-R	ctcgagtgcggccgcaagcttCTAATTTTTAAACTTGAAATCACCTAGGCATG
FcDOH9-F	ccgcgcggcagccatATGCAGAAAATTCCGAAAATTTTTGTG
FcDOH9-R	ctcgagtgcggccgcaagcttCGCTTTAAAATAGTTCAGCAGCATGTTTTC

Note: Uppercase letters indicate homologous arms of the insert fragment; lowercase letters indicate homologous arms of the vector.

Table S8. Strains and plasmids used in this study

Plasmid	Insert fragment	Strain
pET-28a(+)-FcDOH1	G32111	BL21-FcDOH1
pET-28a(+)-FcDOH2	G23279	BL21-FcDOH2
pET-28a(+)-FcDOH3	G11711	BL21-FcDOH3
pET-28a(+)-FcDOH4	G14522	BL21-FcDOH4
pET-28a(+)-FcDOH5	G28014	BL21-FcDOH5
pET-28a(+)-FcDOH6	G8662	BL21-FcDOH6
pET-28a(+)-FcDOH7	G13249	BL21-FcDOH7

Table S9. Activity screening of FcDOH proteins

Substrate	Catalytic Activity	Product
p-Coumaric acid	-	None
Caffeic acid	-	None
Ferulic acid	-	None
Umbelliferone (**1**)	FcDOH2 +	Daphnetin (**6**)
Esculetin (**2**)	-	None
Scopoletin (**3**)	FcDOH2, FcDOH4 +	Esculetin (**2**)
Fraxetin (**5**)	-	None

Esculin (**4**)	-	None

Note: "+" indicates catalytic activity detected; "-" indicates no detectable activity.

Table S10. Primers used for site-directed mutagenesis

primers	Sequences(5'-3')
R221A-F	CTTGTTGTTGCATGCCACTATTATCCAGCTTGTCCAG
R221A-R	GTGGCATGCAACAACAAGCGATTCTGCAC
H223A-F	GTTCGCTGCGCATATTATCCAGCTTGTCC
H223A-R	GGATAATATGCGCAGCGAACAACAAGCGATTC
R155A-F	GCTAACTGGGCTGATTCATTTTACTGCC
R155A-R	GAATCAGCCCAGTTAGCTGCTGGTGAACGATAC
F144A-F	GTAACGCAGATTTGTATCGTTCACCAGC
F144A-R	CAAATCTGCGTTACTAATATAGACTACCGGTC
H240A-F	CACGACCAAGGCCTCAGACAATGATTTC
H240A-R	GTCTGAGGCCTTGGTCGTGCCGAATGTG
T237A-F	CGGCGCTACCAAGCATTCAGACAATG
T237A-R	GAATGCTTGGTAGCGCCGAATGTGAGTTC
R352A-F	CTACTTCGCTGACAAAGGACTTGATGGC
R352A-R	CTTTGTCAGCGAAGTAGGCAGTGTAC
F351A-F	CCTACGCTCGTGACAAAGGACTTGATGGC
F351A-R	CTTTGTCACGAGCGTAGGCAGTGTACTC
D242A-F	CATTCAGCTAATGATTTCATTACAGTGC
D242A-R	GAAATCATTAGCTGAATGCTTGGTCGTG
N243A-F	CATTCAGACGCTGATTTCATTACAGTGC
N243A-R	GAAATCAGCGTCTGAATGCTTGGTCGTG
F312A-F	GTGGCTGCTGCAGTCAGCGCAGGGTC
F312A-R	CGCTGACTGCAGCAGCCACAGAGATTCTTGG

2.Supplementary Figures

Figure S1. BUSCO assessment results of the *F. chinensis* transcriptome assembly

The completeness of three assembly types (trinity.fasta, cluster.fasta, and unigene.fasta) was evaluated using the embryophyta_odb10 database (n = 1,440). c: complete buscos; s: single-copy; d: duplicated; f: fragmented; m: missing.

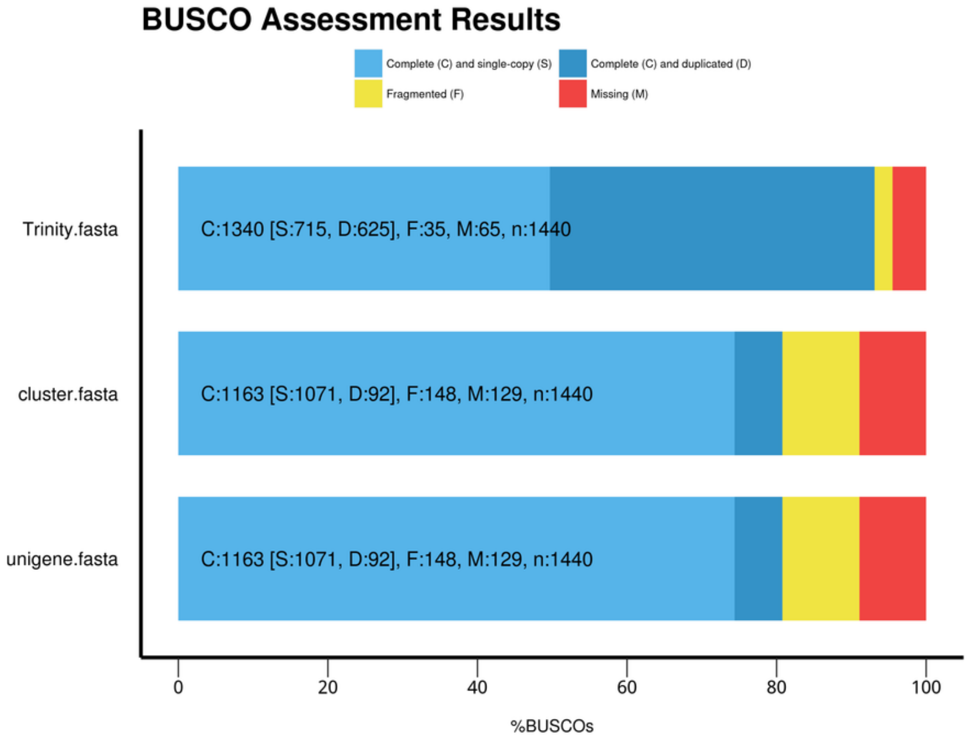

Figure S2. Multiple sequence alignment of candidate FcDOH proteins

The conserved His_1-X-D/E-Xn-His_2 iron-binding motif (black box) and R-X-S 2-OG-binding motif (green box) are indicated. These conserved motifs are present in FcDOH1–FcDOH9 but absent in FcDOH10 and FcDOH11. Multiple sequence alignment was performed using MEGA 12 and visualized with ESPript 3.2.

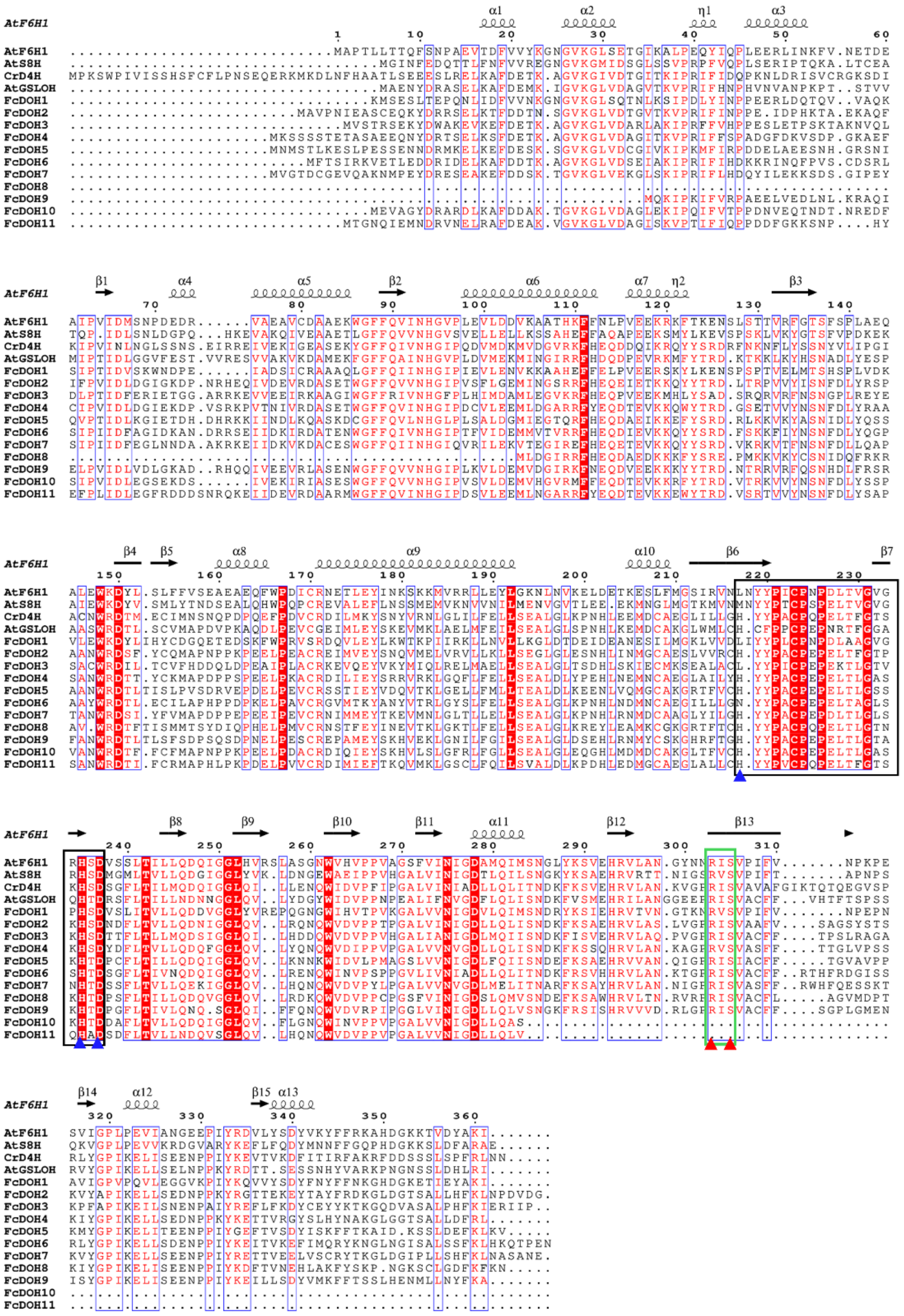
AtF6H1
AtS8H
CrD4H
AtGSLOH
FcDOH1
FcDOH2
FcDOH3
FcDOH4
FcDOH5
FcDOH6
FcDOH7
FcDOH8
FcDOH9
FcDOH10
FcDOH11

Figure S3. SDS-PAGE analysis of recombinant FcDOH proteins

(A) Soluble His-tagged FcDOH proteins. Lanes 1–5: FcDOH2, FcDOH3, FcDOH4, FcDOH5, and FcDOH7, respectively. M: protein molecular weight marker (10–180 kDa). Arrows indicate the target proteins (predicted molecular weight: 42.0–43.1 kDa). (B) Insoluble fraction of FcDOH1. (C) Insoluble fraction of FcDOH6.

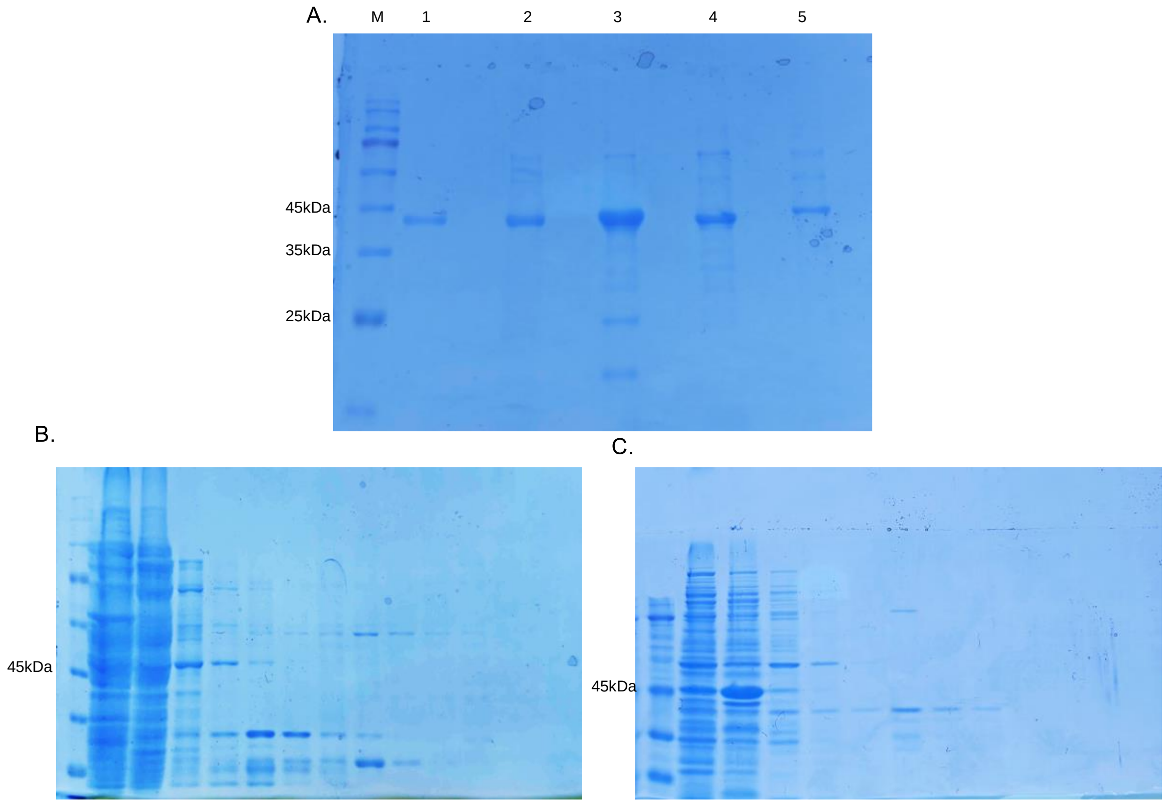

Figure S4. AlphaFold3-predicted structure of FcDOH2

The model is colored by pLDDT confidence scores: very high (pLDDT > 90, blue), confident (90 > pLDDT > 70, light blue), low (70 > pLDDT > 50, yellow), and very low (pLDDT < 50, orange). The core region shows high confidence with pLDDT > 90. The predicted alignment error (PAE) plot is shown on the right, with ipTM = 0.98 and pTM = 0.89, indicating high confidence in the predicted global fold.

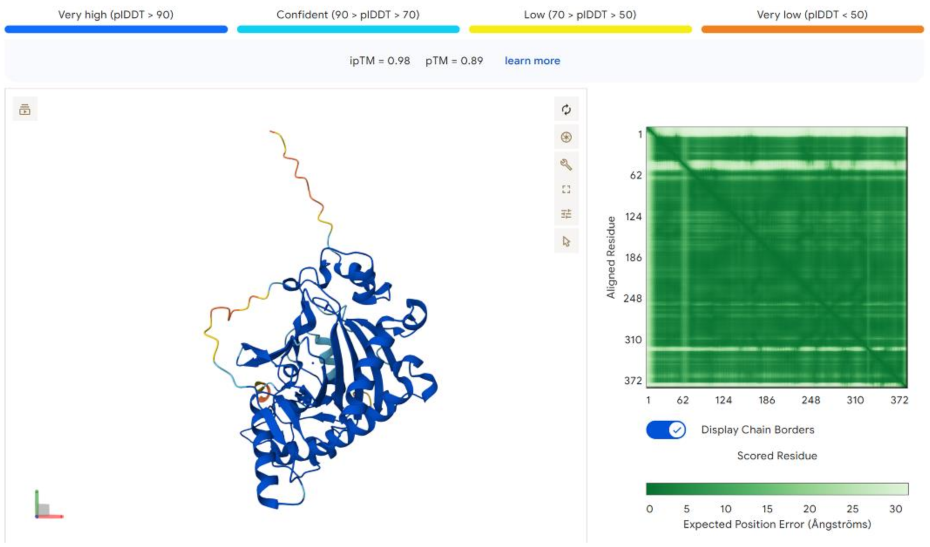

Figure S5. Structural similarity between FcDOH2 and DOXC family proteins with similar functions

Lightorange: FcDOH2; cyan: 4XAE; magenta: 5O7Y.

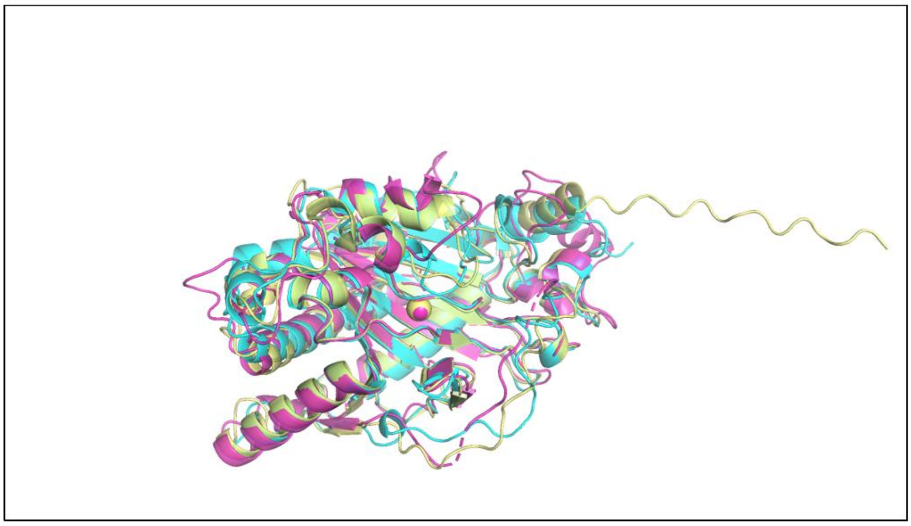

Supplement: Supplementary file 1 [file molecules-31-01787-s001.zip › molecules-4268411-supplementary.pdf]
